# Supplementary material for: Uchimata: a toolkit for visualization of 3D genome structures on the web and in computational notebooks
Source: Bioinformatics. 2026 Jan 22;42(2):btag035. doi: 10.1093/bioinformatics/btag035 (PMC12904833; doi:10.1093/bioinformatics/btag035)
Supplement: btag035_Supplementary_Data [file btag035_supplementary_data.pdf]

Supplemental Materials

for the submission '*Uchimata: a toolkit for visualization of 3D genome structures on the web and in computational notebooks*'

Contents

Code Repositories and Websites.......... 1
3D Genome Visualization Tools: Comparison.......... 2
File Formats Compatibility.......... 4
Challenges of Existing 3D Genome Structure File Formats.......... 4
Incompatible TSV/TXT Example.......... 4
Incompatible PDB Example.......... 5
Uchimata Uses a Standardized Tabular Representation.......... 5
Converting Existing Datasets.......... 6
Converting PDBs from Stevens et al. 2017.......... 6
Converting TSVs from Tan et al. 2018.......... 7
Examples of Uchimata Usage.......... 7
Jupyter Notebooks.......... 8
Inputs Example (examples/jupyter/inputs.ipynb).......... 8
Multiple Structures Example (examples/jupyter/multiple-structures.ipynb).......... 8
Remote Data Example (examples/jupyter/remote-data.ipynb).......... 8
Marimo Notebooks.......... 9
Minimal Example (examples/marimo/minima-example.py).......... 9
Selections Example (examples/marimo/selections.py).......... 9
Compartment Annotation Example (examples/marimo/compartments.py).......... 10
Gene Density Example (examples/marimo/using-bioframe.py).......... 11

Code Repositories and Websites

The two parts of the toolkit (the Javascript library and Python widget) are hosted in separate repositories:

- https://github.com/hms-dbmi/uchimata: Javascript library
- https://github.com/hms-dbmi/uchimata-py: Python widget

Both of these have been archived on Zenodo:

- uchimata: https://doi.org/10.5281/zenodo.17831959
- uchimata-py: https://doi.org/10.5281/zenodo.17832045

Besides these code repositories, the following URL serves as a landing page for the toolkit, and contains a number of interactive notebooks (using the JS library) that highlight the usage of uchimata: https://hms-dbmi.github.io/uchimata/

The auto-generated documentations are hosted within the individual repositories (docs-api/README.md and docs/README.md).

3D Genome Visualization Tools: Comparison

We recognize that direct comparison of features available across available tools is challenging, as capabilities can be hidden behind specific UI or command options. In this table, we make our best attempt at highlighting features that were prioritized in uchimata in order to fill the gap we see among visualization tools for 3D genome structural data.

tool name<br>publication<br>code repository	Software type	Genomics focused	Usable in notebooks	Filtering	Visual encodings
**uchimata**<br>hms-dbmi/uchimata<br>hms-dbmi/uchimata-py	Library / package	Yes ✅	Yes ✅	Genomic ranges, spatial queries (programmatically)	Customizable (declarative specification)
nglutils<br>mirnylab/nglutils	Package	Yes ✅	Yes ✅	No	Fixed (tube)
WashU Epigenome Browser<br>Li 2022, Seng 2025	Genome browser	Yes ✅	No ❌	No	Tube, sphere, cross, line (configurable in GUI), “painting” using additional datasets
Nucleome Browser<br>Zhu 2022	Genome browser	Yes ✅	No ❌	Genomic coordinates via “global / local mode”	Line, stick, cross, sphere. Color by chromosome or additional datasets
Mol*<br>molstar.org<br>molstar/molstar	Library	No ❌	No ❌ (some in development)	Conversion to molecular semantics required (atoms vs bins)	Comprehensive options for typical molecular depictions (incl. line, spheres, balls-and-stick)
PyMOL<br>pymol.org	Library	No ❌	Yes ✅	Conversion to molecular semantics required (atoms vs bins)	Comprehensive options for typical molecular depictions
HiC-3D Viewer<br>Djekidel 2017	Web application	Yes ✅	No ❌	No (but allows highlighting)	Tube, “painting” using additional datasets
CSynth<br>Todd 2020	Modeling engine + viewer	Yes ✅	No ❌	No (but allows highlighting regions	Tube, sphere, line, hull. “Painting” using additional

				between 3D and matrix)	datasets
G3D - https://github.com/lidaof/g3d	Web application	Yes❌	No ❌	Via input field: region, chromosome	Tube, line, sphere. color used for chromosome annotation
SpaceWalk - igvteam/spacewalk	Web application	Yes ✅	No ❌	Genomic ranges based on Hi-C map selections	Points, balls-and-stick
TADkit Serra 2017 (TADbit) 3DGenomes/TADkit	Case-specific genome browser	Yes ✅	No ❌	No	Tube, line, cluster annotation. Color used to show start-end.
HiCube Ye 2023 wmalab/HiCube	Genome browser / web application	Yes ✅	No ❌	Genomic ranges based on Hi-C map selections	Tube/line, sphere for highlighting
Delta Tang 2018 zhangzhwlab/delta	Web application	Yes ✅	No ❌	Rectangular selection via pointer	Line, balls-and-stick
3Disease Browser Li 2016	Case-specific genome browser	Yes ✅	No ❌	No (but allows highlighting regions)	Tube/line, color used to highlight regions

There are also a number of related tools that have been developed and published in the past, but for various reasons we do not consider them to be directly comparable with uchimata. The common reasons are:

- Available only as a **desktop application**: this severely limits integration in bioinformatics workflows and eliminates potential for repurposing or extending the tool; users of such tools rely on importing/exporting all data into/from the desktop tool, and rely on available functionality to do their work.
    - **GLOBE 3D Genome platform** (Knoch 2009): one of the first applications, focuses more on depicting non-spatial genomics data in 3D space
    - **GenomeFlow** (Trieu 2019): a Java-based desktop application
    - **GMOL** (Nowotny 2016): a Java-based desktop application
    - **Genome3D** (Asbury 2010): a C++-based desktop application, to the best of our knowledge the first application to depict 3D models of genomes.
- Tailored toward **AR/VR**: while often intriguing, these platforms also limit integration, require specialized hardware, and can be a barrier in day-to-day analysis.
    - **3DGV** (Zhang 2019): VR application
    - **Delta AR** (Tang 2021): AR application
- **Modeling engines**: although several modeling engines feature visualizations of produced structures, the visualization components are often limited in these large software projects, or they recommend an external tool that serves the visualization

needs.

- **3DGB** (https://github.com/data-fun/3d-genome-builder): modeling engine, does not include visualization capabilities, but recommends Mol* for visualization
- **3D-GNOME** (Wlasnowolski 2023): modeling engine with minor visualization capabilities
- **Chromosome3D** (Adhikari 2016): modeling engine

File Formats Compatibility

Challenges of Existing 3D Genome Structure File Formats

In our work, we encountered a number of formats used to store 3D genome structures. Some of them are adopted from file formats used for protein/molecular data (e.g., PDB, mmcif), while others are more specifically tailored toward genome structures, e.g., g3d.

We see in principle two issues with these existing formats:

1. When molecular formats are used, the authors need to re-interpret the columns tailored for atomistic models for storing attributes relevant to genomic structures (e.g., chromosome and genomic position). In this case, every research group or tool might use different practices, resulting in incompatible file formats despite the files having the same (e.g., .pdb) file extension.
2. Custom file formats suffer from the "N+1" problem and rely on gathering a critical mass of adopters. This, to the best of our knowledge, has not happened for 3D genome structures and rather results in even more incompatibility.

As an illustration of the first problem, we highlight two examples: first a 3D structure stored in a TSV (tab-separated values) format, and a PDB (Protein Data Bank) file format.

Incompatible TSV/TXT Example

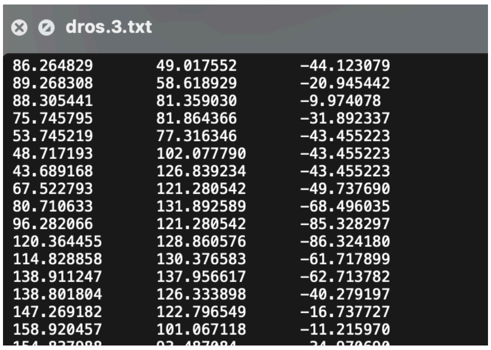

dros.3.txt

```
86.264829	49.017552	-44.123079
89.268308	58.618929	-20.945442
88.305441	81.359030	-9.974078
75.745795	81.864366	-31.892337
53.745219	77.316346	-43.455223
48.717193	102.077790	-43.455223
43.689168	126.839234	-43.455223
67.522793	121.280542	-49.737690
80.710633	131.892589	-68.496035
96.282066	121.280542	-85.328297
120.364455	128.860576	-86.324180
114.828858	130.376583	-61.717899
138.911247	137.956617	-62.713782
138.801804	126.333898	-40.279197
147.269182	122.796549	-16.737727
158.920457	101.067118	-11.215970
```

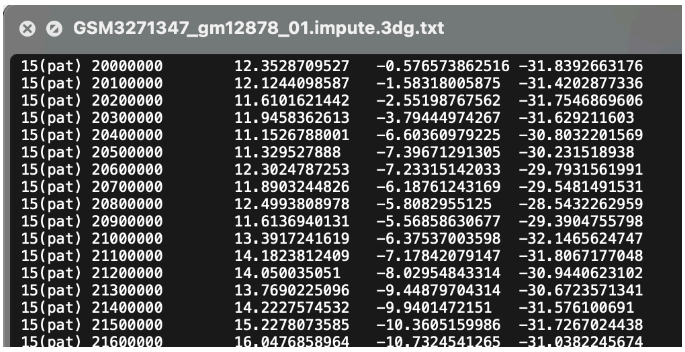

GSM3271347_gm12878_01.impute.3dg.txt

```
15(pat) 20000000	12.3528709527	-0.576573862516	-31.8392663176
15(pat) 20100000	12.1244098587	-1.58318005875	-31.4202877336
15(pat) 20200000	11.6101621442	-2.55198767562	-31.7546869606
15(pat) 20300000	11.9458362613	-3.79444974267	-31.629211603
15(pat) 20400000	11.1526788001	-6.60360979225	-30.8032201569
15(pat) 20500000	11.329527888	-7.39671291305	-30.231518938
15(pat) 20600000	12.3024787253	-7.23315142033	-29.7931561991
15(pat) 20700000	11.8903244826	-6.18761243169	-29.5481491531
15(pat) 20800000	12.4993808978	-5.8082955125	-28.5432262959
15(pat) 20900000	11.6136940131	-5.56858630677	-29.3904755798
15(pat) 21000000	13.3917241619	-6.37537003598	-32.1465624747
15(pat) 21100000	14.1823812409	-7.17842079147	-31.8067177048
15(pat) 21200000	14.050035051	-8.02954843314	-30.9440623102
15(pat) 21300000	13.7690225096	-9.44879704314	-30.6723571341
15(pat) 21400000	14.2227574532	-9.9401472151	-31.576100691
15(pat) 21500000	15.2278073585	-10.3605159986	-31.7267024438
15(pat) 21600000	16.0476858964	-10.7324541265	-31.0382245674
```

An example of two .txt files storing 3D genome structure: (left) a file that separates columns with tabs; (right) a file with the same file extension (.txt) that also includes genomic coordinates. The main problem in this case is that there is no explicit information about which columns encode which data (e.g., what column is the X coordinate or which one is the genomic coordinate), this knowledge is implicitly assumed by the data author and provides a hurdle when it comes to compatibility between tools.

Some other authors or tools could have a different order: for example first storing XYZ coordinates and genomic coordinates in columns after.

Incompatible PDB Example

PDB is very often used to store 3D genome structures. This file format, however, needs to be re-interpretted to capture genomic structures. Duan et al. store the chromosome information in the chain column. Notice how there is no information about the resolution or genomic positions for each bins stored within the file, requiring to supply or assume such information from external sources:

3d_model_of_yeast_genome.pdb

```
ATOM      1 O   EDG A          77.365  61.609  15.448  1.00 75.00
ATOM      2 O   EDG A          78.322  61.414  15.663  1.00 75.00
ATOM      3 O   EDG A          79.279  61.218  15.878  1.00 75.00
ATOM      4 O   EDG A          80.236  61.023  16.093  1.00 75.00
ATOM      5 N   NOD A          76.408  61.804  15.232  1.00 50.00
ATOM      6 O   EDG A          80.762  60.916  16.212  1.00 75.00
ATOM      7 O   EDG A          81.192  60.836  16.314  1.00 75.00
ATOM      8 O   EDG A          81.610  60.777  16.425  1.00 75.00
ATOM      9 O   EDG A          82.018  60.736  16.544  1.00 75.00
ATOM     10 O   EDG A          82.414  60.715  16.673  1.00 75.00
ATOM     11 O   EDG A          82.798  60.713  16.810  1.00 75.00
ATOM     12 O   EDG A          83.172  60.731  16.955  1.00 75.00
ATOM     13 O   EDG A          83.534  60.768  17.109  1.00 75.00
ATOM     14 O   EDG A          83.886  60.824  17.272  1.00 75.00
ATOM     15 O   EDG A          84.225  60.900  17.444  1.00 75.00
ATOM     16 O   EDG A          84.554  60.995  17.624  1.00 75.00
ATOM     17 O   EDG A          84.872  61.110  17.813  1.00 75.00
ATOM     18 O   EDG A          85.178  61.244  18.011  1.00 75.00
ATOM     19 O   EDG A          85.473  61.397  18.217  1.00 75.00
ATOM     20 O   EDG A          85.757  61.569  18.432  1.00 75.00
ATOM     21 O   EDG A          86.029  61.761  18.655  1.00 75.00
```

Stevens et al. on the other hand include the genomic position but include it as an extra column that is non-standard to the PDB standard:

GSM2219497_Cell_1_genome_structure_model.pdb

```
TITLE     Nuc3D genome structure export
REMARK 210
REMARK 210 Atom type C are backbone nodes
REMARK 210 Atom type N are restrained nodes
REMARK 210 Atom number increases every 100000 bases
REMARK 210 Residue code indicates chromosome
REMARK 210 Residue number represents which sequence Mb the atom is in
REMARK 210 Chain letter is different every chromosome, where Chr1=a, Chr2=b etc.
REMARK 210 Extended PDB format with particle seq. pos. in last column
REMARK 210
MODEL        1
HETATM    1  N1  chr a   4       9.938  -6.486  -1.413  0.00  1.00           N     3000000
HETATM    2  N2  chr a   4       9.186  -7.260  -0.924  0.00  1.00           N     3100000
HETATM    3  N3  chr a   4       8.522  -7.236  -0.530  0.00  1.00           N     3200000
HETATM    4  N4  chr a   4       7.598  -6.794  -1.174  0.00  1.00           N     3300000
HETATM    5  N5  chr a   4       6.961  -6.321  -0.707  0.00  1.00           N     3400000
HETATM    6  N6  chr a   4       7.303  -5.744   0.358  0.00  1.00           N     3500000
HETATM    7  N7  chr a   4       7.471  -5.079  -0.570  0.00  1.00           N     3600000
HETATM    8  N8  chr a   4       7.653  -4.782  -1.280  0.00  1.00           N     3700000
HETATM    9  N9  chr a   4       8.339  -5.284  -1.892  0.00  1.00           N     3800000
HETATM   10  N10 chr a   4       9.051  -6.114  -2.713  0.00  1.00           N     3900000
```

In this case, REMARK records are used to explain the interpretation of columns, which, however, requires manual interpretation. The chromosome names are also encoded with letters and need to be explicitly decoded to be linked with other genomics data for further analysis.

Similar looseness exists in other formats which makes implementing a universal data loader for these file formats difficult, if not impossible.

Uchimata Uses a Standardized Tabular Representation

For the above mentioned reasons, uchimata does not implement loaders for PDBs, XYZs, or similar files directly. Instead, we realize that **3D genome structures represent yet another tabular data format** like many others in bioinformatics, and that **there exist standardized**

and widely used formats to store such data. Such formats have been developed and adopted by broader communities beyond genomics or bioinformatics, which has led to more engineering effort and tooling available for working with such data. This is why at the core Javascript library, we use the Apache Arrow standard. This is inspired by and aligned with other efforts in bioinformatics tools: see the Oxbow project (github, blog post), which provides a package that unifies many of genomics file formats into an Arrow representation. This also makes it easy to convert to the DataFrame structure which is commonly used for data wrangling during bioinformatics analysis.

That being said, we acknowledge that it is essential to show how researchers can load existing datasets that are stored in other formats, such as the mentioned .PDB or .XYZ files. For this reason, we provide examples for how a number of file formats can be converted for ingestion by uchimata, described in the next section of this document.

Converting Existing Datasets

We provide scripts that show conversion of structures from the Stevens et al. and Tan et al. publications. Both output Arrow files that can be directly used as input for the Javascript library. From the Arrow representation, it is also very simple to turn these structures into Python-native structures, such as pandas DataFrame, which can then be used for downstream analysis and processing in a notebook. Dataframes can be also directly inputted into the uchimata Python widget.

Converting PDBs from Stevens et al. 2017

(folder: `uchimata-py/data/stevens-2017`)

GSM2219497_Cell_1_genome_structure_model.pdb

```
TITLE     Nuc3D genome structure export
REMARK 210
REMARK 210 Atom type C are backbone nodes
REMARK 210 Atom type N are restrained nodes
REMARK 210 Atom number increases every 100000 bases
REMARK 210 Residue code indicates chromosome
REMARK 210 Residue number represents which sequence Mb the atom is in
REMARK 210 Chain letter is different every chromosome, where Chr1=a, Chr2=b etc.
REMARK 210 Extended PDB format with particle seq. pos. in last column
REMARK 210
MODEL        1
HETATM    1  N1  chr a   4       9.938  -6.486  -1.413  0.00  1.00           N      3000000
HETATM    2  N2  chr a   4       9.186  -7.260  -0.924  0.00  1.00           N      3100000
HETATM    3  N3  chr a   4       8.522  -7.236  -0.530  0.00  1.00           N      3200000
HETATM    4  N4  chr a   4       7.598  -6.794  -1.174  0.00  1.00           N      3300000
HETATM    5  N5  chr a   4       6.961  -6.321  -0.707  0.00  1.00           N      3400000
HETATM    6  N6  chr a   4       7.303  -5.744   0.358  0.00  1.00           N      3500000
HETATM    7  N7  chr a   4       7.471  -5.079  -0.570  0.00  1.00           N      3600000
HETATM    8  N8  chr a   4       7.653  -4.782  -1.280  0.00  1.00           N      3700000
HETATM    9  N9  chr a   4       8.339  -5.284  -1.892  0.00  1.00           N      3800000
HETATM   10  N10 chr a   4       9.051  -6.114  -2.713  0.00  1.00           N      3900000
HETATM   11  N1  chr a   5       8.203  -6.855  -2.195  0.00  1.00           N      4000000
HETATM   12  N2  chr a   5       7.377  -7.196  -1.610  0.00  1.00           N      4100000
HETATM   13  N3  chr a   5       7.980  -7.995  -0.746  0.00  1.00           N      4200000
HETATM   14  N4  chr a   5       8.197  -8.943  -0.472  0.00  1.00           N      4300000
HETATM   15  C5  chr a   5       8.424  -9.302   0.485  0.00  1.00           C      4400000
HETATM   16  N6  chr a   5       9.586 -10.127   2.636  0.00  1.00           N      4500000
HETATM   17  N7  chr a   5       9.884 -10.486   2.403  0.00  1.00           N      4600000
HETATM   18  N8  chr a   5      10.293 -11.251   1.837  0.00  1.00           N      4700000
HETATM   19  N9  chr a   5      10.687 -11.387   0.676  0.00  1.00           N      4800000
```

Example code for converting structures in a PDB format can be found in the uchimata-py repository, under the `data/stevens-2017` folder. As the name suggests, this data comes from the Stevens 2017 publication. We downloaded the data deposited on GEO: GSE80280, and copied over only the structures (identified by the .pdb extension). For this demonstration, we only selected a single file as an example, to prevent storing large amounts of data in a Github repository. The caveat of this specific dataset is that each .pdb file holds a number of structures. The script in `notebook.ipynb` extracts these multiple models into separate Arrow files, and stores them in the `out` subfolder.

Converting TSVs from Tan et al. 2018

(folder: `uchimata-py/data/tan-2018`)

GSM3271347_gm12878_01.impute.3dg.txt

15(pat)	20000000	12.3528709527	-0.576573862516	-31.8392663176
15(pat)	20100000	12.1244098587	-1.58318005875	-31.4202877336
15(pat)	20200000	11.6101621442	-2.55198767562	-31.7546869606
15(pat)	20300000	11.9458362613	-3.79444974267	-31.629211603
15(pat)	20400000	11.1526788001	-6.60360979225	-30.8032201569
15(pat)	20500000	11.329527888	-7.39671291305	-30.231518938
15(pat)	20600000	12.3024787253	-7.23315142033	-29.7931561991
15(pat)	20700000	11.8903244826	-6.18761243169	-29.5481491531
15(pat)	20800000	12.4993808978	-5.8082955125	-28.5432262959
15(pat)	20900000	11.6136940131	-5.56858630677	-29.3904755798
15(pat)	21000000	13.3917241619	-6.37537003598	-32.1465624747
15(pat)	21100000	14.1823812409	-7.17842079147	-31.8067177048
15(pat)	21200000	14.050035051	-8.02954843314	-30.9440623102
15(pat)	21300000	13.7690225096	-9.44879704314	-30.6723571341
15(pat)	21400000	14.2227574532	-9.9401472151	-31.576100691
15(pat)	21500000	15.2278073585	-10.3605159986	-31.7267024438
15(pat)	21600000	16.0476858964	-10.7324541265	-31.0382245674
15(pat)	21700000	15.9320806361	-10.6769482659	-29.921840112
15(pat)	21800000	14.9828990876	-10.431802412	-29.4055623053
15(pat)	21900000	14.2452058267	-9.76349858723	-28.8993015406
15(pat)	22000000	12.8837634415	-8.58957202898	-28.557413499
15(pat)	22100000	14.9774772582	-8.33059114718	-29.7166507221
15(pat)	22200000	15.6683232391	-7.76195992652	-30.7330327562
15(pat)	22300000	15.9086409774	-6.58606466259	-31.045342132
15(pat)	22400000	15.4705810466	-5.66566613757	-30.4482014144
15(pat)	22500000	13.5412419099	-5.41155068141	-28.998925583
15(pat)	22600000	12.4139927656	-6.423350512	-28.5680751598
15(pat)	22700000	12.6754685975	-7.1979813889	-27.2395838814
15(pat)	22800000	13.824155854	-8.19786986784	-26.4292874617
15(pat)	22900000	14.1322251989	-8.99233987883	-25.1266515095

A second example uses the structures published with the Tan et al. 2018 paper. We similarly downloaded the data from GEO: GSE117876, identified the 3D genome structures (.3dg.txt extension). We again only provide a single input structure in this demonstration, to minimize use of storage in the Github repository. This dataset uses a tab-separated format. Interestingly, the authors chose to encode the maternal/paternal chromosome association in the chromosome column, although this could have been an additional field. Nevertheless, it is easy to extract this value into a separate column afterward.

Examples of Uchimata Usage

To better illustrate the capabilities of uchimata, in this part of the Supplemental Materials we give short descriptions of the examples for using the toolkit to visualize 3D genome structures that are available in the Github repositories. We focus on the uchimata Python package (uchimata-py), as it more closely relates to the usage in biologically relevant cases.

Jupyter Notebooks

Jupyter notebooks represent the conventional workbench used daily by many computational biologists.

Inputs Example (`examples/jupyter/inputs.ipynb`)

This example notebook shows various ways of supplying the input data: 1) loading from a .arrow file from disk, 2) passing a numpy array, 3) parsing an .xyz string. We lean into the compositional nature of Python and notebooks, we show how libraries such as numpy and pyarrow are used to wrangle data. Another aspect showcased by this example is the conversion of an .xyz file that stores 3D structure coordinates. We use the pyarrow package to transform this file into an Arrow Table which then gets passed to uchimata.

Multiple Structures Example (`examples/jupyter/multiple-structures.ipynb`)

This example showcases the ability to display multiple structures in one widget. Each structure can have its own view config (and therefore different visual appearance). The structure used here is a mock random walk, represented as a numpy array. We filter the array to grab a small substructure, which we highlight using a red color and a slightly larger mark size.

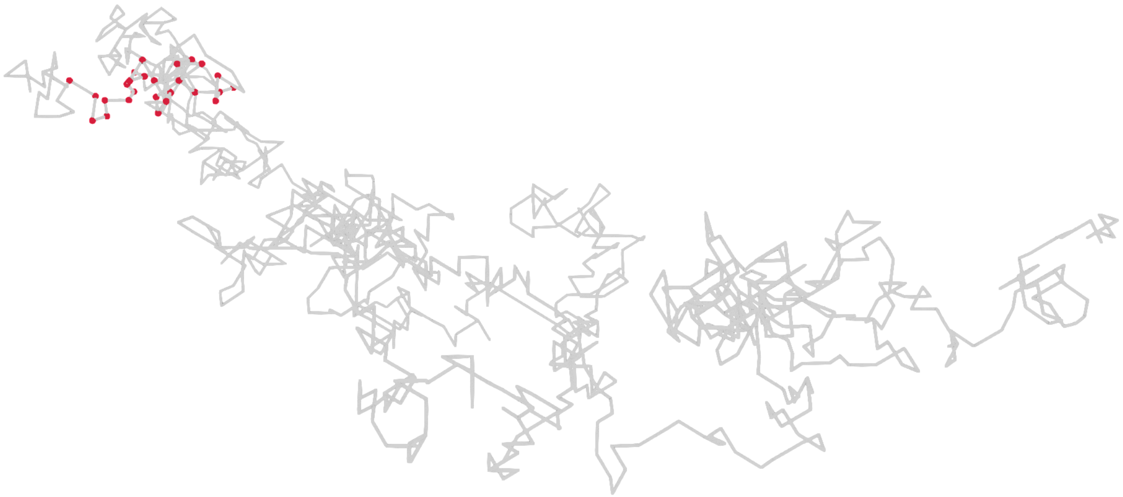

Remote Data Example (`examples/jupyter/remote-data.ipynb`)

A very simple example that shows how users might fetch an Arrow file stored in the cloud. In this example, we use the requests package to download an .arrow file containing a 3D genome structure from Cloudflare R2 storage. Such structure can be generated by scripts for conversion described above.
This example shows the universality of sharing the same datafiles between the Python widget and the Javascript library, as the same file can be consumed by both. This is primarily enabled by the proliferation of Apache Arrow as a standard, as there exist referential implementations for working with this standard in many programming languages.

Marimo Notebooks

Marimo notebooks are a new type of computational notebooks based on Python. Their key feature is the reactivity of variables defined across cells, which makes it easy to build simple interactive visualizations.

Minimal Example (`examples/marimo/minima-example.py`)

This example does not require an input file, instead, it generates a mock structure using a random walk, in the form of a two-dimensional NumPy array, which can be directly visualized using uchimata. Here we also demonstrate how the view configuration structure can be used to map an array of values—in this case indices for the bins—to a color visual channel. This type of declarative visual encoding specification is inspired by the [Grammar of Graphics]() approach.

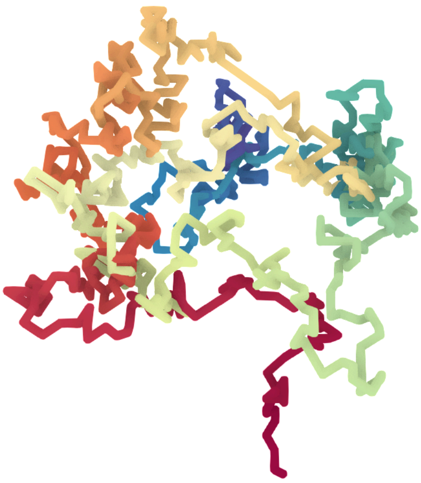

Selections Example (`examples/marimo/selections.py`)

This notebook demonstrates basic genomic selections. From the whole genome structure:

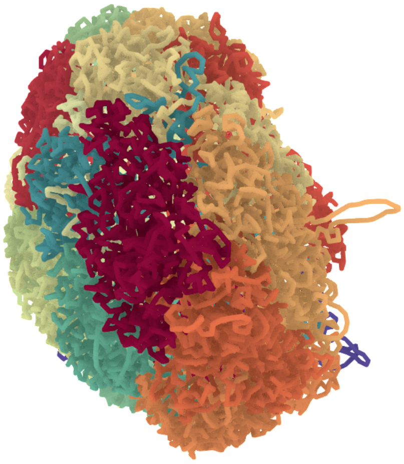

users can either select a specific chromosome:

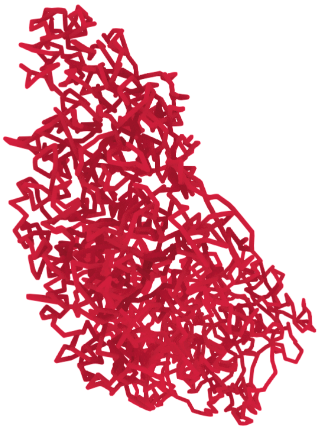

```python
# Selecting via chromosome names
model_chr1 = uchi.select(model, "chr a")
w3 = uchi.Widget(model_chr1, viewconfig={"color": "crimson", "links": True})
```

or use a bioframe dataframe to define selected ranges:

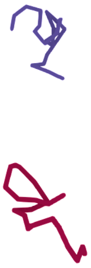

```python
## Using a dataframe created via `bioframe`

# The chromosome names ("chr a", "chr b") might seen unusual, but it is simply taken verbatim from what the authors store in the PDB with the publication
df3 = bioframe.from_any(
    [['chr a', 3000000, 5000000],
     ['chr b', 3000000, 5000000]],
    name_col='chrom')

submodel = uchi.select_bioframe(model, df3)
w2 = uchi.Widget(submodel, viewconfig=vc1)
```

Compartment Annotation Example (`examples/marimo/compartments.py`)

This example showcases how the declarative visual encoding specification can be used to create arbitrary annotation. In this example, we load an external dataset (in the form of a .csv file) into a dataframe in a notebook. We do some processing to match resolution of the 3D structure and then use bioframe to merge the two dataframes. Uchimata is then used for visualization, where the resulting dataframe is directly supplied as input to the widget.

index<br>int64	chrom<br>object<br>unique: 1	start<br>int64	end<br>int64	comp_name<br>object<br>unique: 228	comp_rank<br>float64	continous_rank<br>float64
0	chr16	0	100000	A.1.1.1.2.2.1	1	0.9736842105
10	chr16	100000	200000	A.1.1.1.2.2.1	1	0.9736842105
20	chr16	200000	300000	A.1.1.1.2.2.1	1	0.9736842105
30	chr16	300000	400000	A.1.1.1.2.2.1	1	0.9736842105
40	chr16	400000	500000	A.1.1.1.2.2.1	1	0.9736842105
50	chr16	500000	600000	A.1.1.1.2.2.1	1	0.9736842105
60	chr16	600000	700000	A.1.1.1.2.2.1	1	0.9736842105
70	chr16	700000	800000	A.1.1.1.2.2.1	1	0.9736842105
80	chr16	800000	900000	A.1.1.2.2.2.2.1.2	1	0.9298245614
90	chr16	900000	1000000	A.1.1.2.2.2.2.1.2	1	0.9298245614

We use the viewconfig parameter to specify that the color of the bin marks (spheres here) will be determined based on the ‘comp_name’ column in the supplied dataframe:

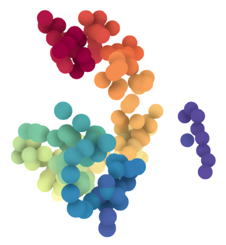

```
vc = {
    "color": {

        "field": "comp_name",
        "colorScale": "Spectral"
    },
    "scale": 0.04, "links": False, "mark": "sphere"
}

w_final = uchi.Widget(merged_df, viewconfig=vc)
w_final
```

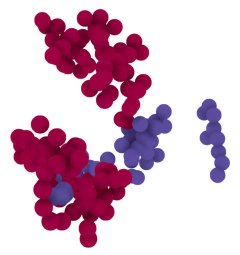

```
vc2 = {
    "color": {
        "field": "comp_name",
        "colorScale": "Spectral"
    },
    "scale": 0.04, "links": False, "mark": "sphere"
}

w_final2 = uchi.Widget(merged_df_only_AB, viewconfig=vc2)
w_final2
```

Left: uses the subcompartments (e.g., A.1.1.1.2.2.1), right: only shows the highest level of compartment annotation (A or B).

Gene Density Example (`examples/marimo/using-bioframe.py`)

This example demonstrates how users might employ uchimata in correlating genomic data with the 3D genome structures. We are using the structures from Stevens et al. 2017, and fetch appropriate gene annotations for the mm10 assembly. We use bioframe to load the GTF file to a dataframe structure. Thanks to representing the 3D structure in the Arrow tabular format, it is easy to transform the Arrow to a dataframe as well. Afterward, we use functions of bioframe to join the two dataframe and calculate

We display the 3D structure and map the gene density values to the color:

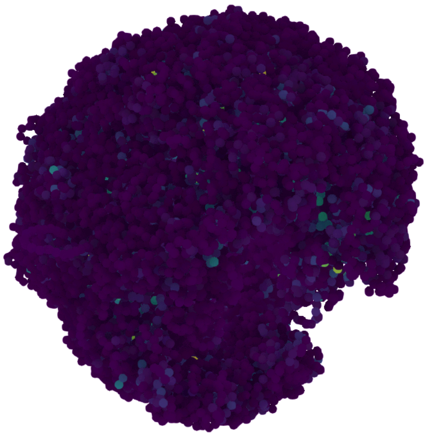

By using a different color map and adding a cutting plane filter, we can produce the following visualization, which gives us the insight that gene rich regions lie within the nuclear space, further from the borders of the genome structure:

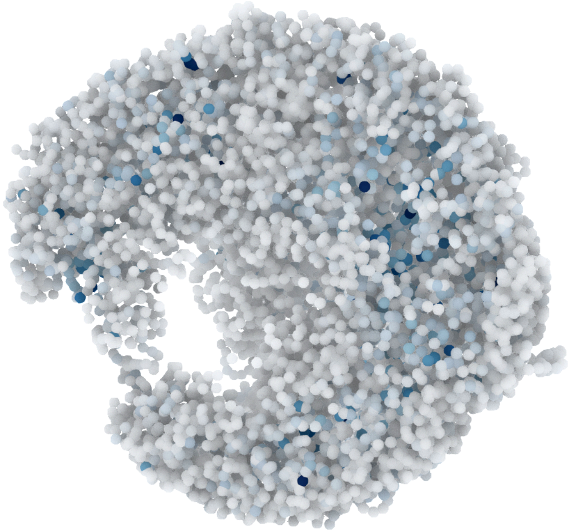

Final step is to map the gene density values to both the color and the scale of the bin marks, which gets rid of the occlusion to highlight gene rich regions hidden within the structure:

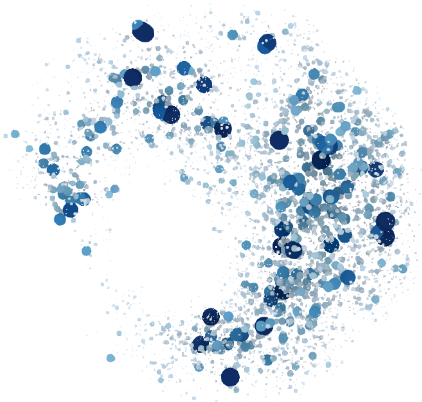

The point of the uchimata toolkit is to enable creation of visualizations like these to develop or confirm insights. The tool aims to be flexible, customizable, and within reach during the analysis.
